# HIV Pre-Exposure Prophylaxis Awareness among Individuals in Treatment for Opioid Use Disorder in the United States: A Cross-Sectional Study

**DOI:** 10.1007/s10461-026-05057-x

**Published:** 2026-03-17

**Authors:** Mance E. Buttram, Matthew S. Ellis, José F. Colón Burgos

**Affiliations:** 1 Department of Health, Human Performance, and Recreation, University of Arkansas, Fayetteville, AR USA; 2 Center for Public Health and Technology, University of Arkansas, Fayetteville, AR USA; 3 Department of Psychiatry, Washington University in St. Louis School of Medicine, St. Louis, MO USA; 4 Department of Environmental and Global Health, College of Public Health and Health Professions, University of Florida, Gainesville, USA

**Keywords:** PrEP awareness, Opioid use disorder, Methadone, HIV, Opioid treatment

## Abstract

This study examined PrEP awareness among HIV-negative individuals in treatment for opioid use disorder (OUD; *n* = 179), and associations with demographic characteristics, sexual and injection behaviors, and treatment modalities. Overall, 48% of participants reported PrEP awareness. Bivariate logistic regression showed that, compared to others, individuals who were older, African American/Black, and in methadone treatment less frequently reported PrEP awareness (*p*≤.018), while those who reported sex with a person who injects drugs, inpatient/day treatment, and self-help group attendance more frequently reported PrEP awareness (*p*≤.041). Increased education and outreach to PrEP eligible individuals in methadone and other opioid treatment programs appears warranted.

## Introduction

Pre-exposure prophylaxis (PrEP) can reduce the risk of acquiring HIV through sex and among people who inject drugs (PWID) by 90% and 70%, respectively [1]. Since initial PrEP guidance was published by the U.S. Centers for Disease Control and Prevention (CDC), awareness and uptake of PrEP has grown, with 36% of PrEP eligible individuals in the U.S. receiving a prescription in 2022 [2]. Data also indicate that among PWID, PrEP awareness increased from 25.6% in 2018 to 35.3% in 2022; however, uptake only reached 1.2% [3]. Yet, PWID, including opioids, can also have heightened risk for HIV transmission related to injection and sexual behaviors [4].

In response, multiple studies have examined potential avenues to increase PrEP awareness and uptake in existing health and social service settings utilized by people who use drugs. These include addiction consultations in hospitals, syringe service programs, homeless support organizations, and treatment for opioid use disorder (OUD) [5]. Yet, additional data are needed to assess multi-level factors associated with overlooked populations who may benefit from PrEP [5]. The aim of this brief report is to contribute to addressing this deficit by examining data on PrEP awareness among individuals in treatment for OUD, and associations with individual (e.g., demographic characteristics, sexual and injection behaviors) and organizational (e.g., treatment modalities) social ecological factors.

## Methods

Data for this secondary analysis are drawn from a study examining social ecological factors related to the non-medical use of gabapentin in combination with opioids. Eligible individuals were ≥ age 18 and reported use of illicit opioids (i.e., heroin; fentanyl) and/or non-medical use of prescription opioids (e.g., oxycodone; hydrocodone) in the 90 days prior to treatment entry. All HIV-negative individuals were included in the analysis. The total sample (*n* = 179) included participants who reported gabapentin NMU in the past year (*n* = 150) with the remaining participants reporting gabapentin use only as prescribed (*n* = 29).

Participants were recruited through OUD treatment centers. There were 21 treatment centers which received flyers and advertisements to post in public spaces (e.g., waiting area; front desk). Recruitment materials directed interested individuals to call, e-mail, or use a QR code to access additional information and complete the eligibility screener. Eligible participants were contacted and completed survey interviews via Zoom. In addition, eight treatment centers allowed the research team to visit, announce the study, conduct eligibility screenings, and administer surveys.

Prior to each interview, participants provided informed consent and were re-screened for eligibility. Interviews lasted 60–90 min. Participants received a $50 gift card for their participation. All study procedures were approved by the university’s Institutional Review Board.

The survey was based on the Global Appraisal of Individual Needs (GAIN), which includes sections on demographic information, substance use, sexual behaviors, and physical and mental health [6]. As part of the survey, participants were provided with a definition of PrEP, “Taking a medication on a daily basis to reduce or prevent getting HIV infection,” and were then asked, “Have you ever heard of PrEP before?”

Participants reported their age. Race/ethnicity was assessed by asking participants whether they were Hispanic or Latino/a, followed by asking them what race/ethnicity they consider themselves to be. Participants reported sexual orientation. Responses other than heterosexual/straight (e.g., gay; lesbian; bisexual; something else) were coded as sexual minority. Years of education was assessed with the question, “What is the last grade or year that you completed in school?” Participants were asked to report sexually transmitted infection (STI) diagnoses of syphilis, chlamydia, and gonorrhea, which were combined into STI history.

Sexual and injection behaviors were assessed by asking participants to report the total number of vaginal and anal sex partners in the 90 days prior to treatment. Participants who reported two or more sex partners and either no primary partner or a non-monogamous primary partner were coded as having multiple non-monogamous sex partners. Additional items inquired about past 12-month behaviors including, “Did you have sex to get drugs, gifts, or money?” (i.e., trade/sell sex), “Did you use drugs, gifts, or money to purchase or get sex?” (i.e., purchase sex), and “Have you ever had sex with a person who injects drugs?” (i.e., sex with PWID). Participants were also asked if they had ever participated in group sex. Individuals reporting injection drug use (IDU) in the past 12 months, were asked, “Have you ever used a needle that you knew or suspected someone else had used before,” and “Have you ever used a needle without cleaning it”?

Participants were asked to report the type of treatment they were receiving: residential, inpatient/day treatment, outpatient, working with a counselor or case worker, buprenorphine prescription, or enrolled in a methadone program. Although participants could select all applicable, responses were categorized based on the most intensive treatment modality in which a participant interacts with treatment professionals. For example, if a participant reported residential treatment and receiving buprenorphine, the response was coded as residential treatment. Codes for each response were agreed upon by all authors. Finally, participants reported if they attended a self-help group meeting such as Narcotics Anonymous in the 90 days prior to treatment.

Bivariate logistic regressions were conducted to examine the odds of PrEP awareness among variables of interest. The Holm-Bonferroni method was used to control for multiple bivariate tests. Analyses were conducted via SPSS ver. 27.

## Results

In total there were 179 HIV-negative participants from 23 states who were eligible and included in analyses. 48% of participants reported awareness of PrEP. Overall, participants identified as female (43.6%), Hispanic (26.8%), African American/Black (13.4%), white (46.4%), Native American (10.1%), other race/ethnicity (3.4%), and sexual minority (11.7%). Mean age was 41.1 and mean years of education was 12.3.

Results of regression analyses are shown in Table 1. All findings remained significant after Holm-Bonferroni adjustment. For each additional year of age, the likelihood of reporting PrEP awareness decreased (OR = 0.949, 95% CI = 0.920-0.978). Findings related to race/ethnicity were largely not significant, however, African American/Black participants had approximately 69% lower odds of reporting PrEP awareness, compared to those of other races/ethnicities (OR = 0.308, 95% CI = 0.116-0.819). Individuals who reported having sex with a person who injects drugs were more than twice as likely to report PrEP awareness (OR = 2.386, 95% CI = 1.272–4.476).

**Table 1:** Bivariate logistic regression models of background characteristics, sexual and injection behaviors, and treatment for substance use disorder associated with PrEP awareness among individuals in treatment for OUD (*N* = 179)

	PrEP Aware		Not Aware		*p*	OR	95% CI
	*N* = 86	48.0%	*N* = 93	52.0%			
Demographics and Background							
Age					0.001	0.949	0.920, 0.978
Female	38	44.2%	40	43.0%	0.874	1.049	0.581, 1.895
Hispanic	25	29.1%	23	24.7%	0.541	1.230	0.634, 2.386
African American/Black	6	7.0%	18	19.4%	0.018	0.308	0.116, 0.819
White	45	52.3%	38	40.9%	0.142	1.560	0.862, 2.821
Native American	7	8.1%	11	11.8%	0.552	0.735	0.267, 2.027
Other race/ethnicity	3	3.5%	3	3.2%	0.922	1.084	0.213, 5.523
Sexual minority	14	16.3%	7	7.5%	0.075	2.389	0.915, 6.238
Education ≥ 12 years	74	86.0%	78	83.9%	0.548	1.294	0.558, 2.998
STI history	25	29.1%	29	31.2%	0.396	0.765	0.412, 1.420
Sexual and Injection Behaviors							
Multiple sex partners	26	30.2%	24	25.8%	0.510	1.246	0.648, 2.396
Buy sex	12	14.0%	6	6.5%	0.103	2.351	0.841, 6.572
Trade/sell sex	13	15.1%	6	6.5%	0.067	2.582	0.935, 7.133
Group sex history	30	34.9%	26	28.0%	0.319	1.380	0.732, 2.602
Sex with person who injects drugs	39	45.3%	24	25.8%	0.007	2.386	1.272, 4.476
Use a needle someone else used had used before	17	19.8%	9	9.7%	0.391	1.539	0.575, 4.120
Use a needle without cleaning it	15	17.4%	9	9.7%	0.644	1.264	0.467, 3.420
Treatment and Support for Substance Use Disorder							
Buprenorphine treatment	4	4.7%	3	3.2%	0.625	1.463	0.318, 6.735
Methadone treatment	29	33.7%	50	53.8%	0.007	0.438	0.239, 0.801
Residential treatment	21	24.4%	18	19.4%	0.413	1.346	0.661, 2.743
Inpatient/day treatment	24	27.9%	13	14.0%	0.024	2.382	1.123, 5.053
Outpatient treatment	4	4.7%	7	7.5%	0.428	0.599	0.169, 2.124
Counseling or case worker	4	4.7%	2	2.2%	0.365	2.220	0.396, 12.437
Self-help group attendance	39	45.3%	28	30.1%	0.041	1.897	1.026, 3.506

Regarding treatment and support for OUD, there were several findings related to treatment modality. Compared to participants in other treatment modalities, those in methadone treatment had approximately 56% lower odds of reporting PrEP awareness (OR = 0.438, 95% CI = 0.239-0.801), while those in inpatient/day treatment were more than twice as likely to do so (OR = 2.382, 95% CI = 1.123–5.053). In addition, participants who attended a self-help group meeting in the 90 days before treatment were more likely to report PrEP awareness (OR = 1.897, 95% CI = 1.026–3.506).

## Discussion

Overall, in this sample of individuals in treatment for OUD, most of which also reported gabapentin NMU, approximately half noted PrEP awareness. Few studies of OUD treatment clients report gabapentin NMU, yet for context, this is higher than 5%-18% awareness reported in a recent review which included samples of people in treatment for OUD [7], and lower than the 67% recently reported from an urban methadone clinic [8].

Multiple sexual and injection behaviors, which may place individuals at risk for acquiring HIV infection, were reported by over half of the total sample, including having multiple, non-monogamous sex partners, group sex history, and having sex with a PWID. Additionally, large pluralities of participants also reported trading/selling sex and engaging in drug injection with a previously used or unclean needle. Thus, it appears many participants would likely be eligible for PrEP initiation. In fact, studies have documented that risk for HIV is reduced once individuals enter OUD treatment and initiate methadone or buprenorphine [9]. Thus, efforts to increase PrEP awareness and uptake among individuals in OUD treatment must be a priority.

Findings illustrate that methadone treatment clients less frequently report PrEP awareness, compared to individuals in other treatment modalities. This is noteworthy, considering that much attention has been given to opioid treatment programs (OTPs) and the potential to include PrEP inside these settings [8, 10]. Federal guidelines require OTPs to perform physical and behavioral health assessments for incoming patients, develop patient-centered care plans, and provide counseling [11]. Such an environment would be conducive to increasing PrEP awareness, acceptability, and uptake. Although it is recommended that OTP patients are evaluated for PrEP, a recent survey found that just 61% of OTPs provide HIV testing and only 9.5% offer PrEP [12]. As noted in a recent review, clinical settings, including OTPs, often do not have the infrastructure or capacity to address PrEP, which creates a barrier for integrated care [5].

Implementing PrEP services in OTPs and other treatment settings would address some barriers in one’s social environment, yet others remain, including cost, competing priorities among clients, stigma (including intersectional stigmas) against PWUD and people with heightened risk for HIV infection, as well as medical mistrust among African American/Black and other marginalized populations [5, 10]. This is especially important, given that African American/Black populations may have increased risk for HIV and yet may not be provided with PrEP information [5]. Yet, the existing structure and resources of OTPs would likely be an ideal setting for increasing access to PrEP for people with OUD. Continuing research is needed to examine barriers and facilitators for implementing PrEP inside methadone clinics. Such data would be informative, given the changing nature of OTPs which can include take-home medications, of which the impact on PrEP awareness, acceptability, and uptake is not apparent. Furthermore, elucidating such barriers would aid in the development and testing of implementation studies to strengthen linkages to PrEP services and HIV care through partnerships between OTPs and local health departments or federally qualified healthcare centers.

There are some limitations which must be noted. Because of the eligibility criteria, which included recent opioid use, treatment admission, and use of gabapentin (either as prescribed or non-medical use), generalizability to other populations of PWUD or people in treatment for OUD is limited. The total sample, which included a primary sample of individuals who non-medically used gabapentin, and a smaller number of individuals who used only as prescribed, further limits generalizability. These data are based on self-report and data can be subject to recall and social desirability bias, as well as interviewer effects. The coding of treatment modality limits the ability to explore nuances of overlapping treatment exposures. Finally, future work should engage in robust multivariable analyses with a focus on intersectional vulnerabilities and other factors which were outside the scope of this secondary analysis.

In spite of these limitations, findings presented in this brief report show that nearly half of individuals receiving treatment for OUD report PrEP awareness. More work needs to be done, especially for those who would appear to meet PrEP eligibility. Increased education and outreach in OTPs, including methadone clinics, appears warranted.

## Data Availability

Data are available by request.

## References

[1] Chou R, Evans C, Hoverman A, Sun C, Dana T, Bougatsos C, Grusing S, Korthuis PT. (2019). Preexposure prophylaxis for the prevention of HIV infection: evidence report and systematic review for the US Preventive Services Task Force. JAMA. 2019;321(22):2214–2230. 10.1001/jama.2019.2591 31184746

[2] Fanfair RN, Mermin J. Expanding PrEP coverage in the United States to achieve the EHE goals. National Center for HIV, Viral Hepatitis, STD, and Tuberculosis Prevention, Centers for Disease Control and Prevention; 2023. Available at https://www.cdc.gov/nchhstp/director-letters/expanding-prep-coverage.html. Accessed May 6, 2025.

[3] Chapin-Bardales D, Broz D, Eustaquio P et al. Changes in HIV PrEP awareness and use among PWID in 19 US Cities, 2018 and 2022. Conference on Retroviruses and Opportunistic Infections (CROI); 2024; Denver, Colorado, United States.

[4] Handanagic S, Finlayson T, Burnett JC, Broz D, Wejnert C. HIV infection and HIV-associated behaviors among persons who inject drugs — 23 metropolitan statistical areas, united States, 2018. MMWR Morb Mortal Wkly Rep. 2021;70:1459–65. 34673746 10.15585/mmwr.mm7042a1 PMC9361835

[5] McCoy K, Mantell JE, Deiss R, Liu A, Bauman LJ, Bonner CP, Vinson J, Buchbinder S. Pre-exposure prophylaxis awareness and demand creation: overlooked populations and opportunities to move forward. J Acquir Immune Defic Syndr. 2025;98(5S):e170–80. 40163069 10.1097/QAI.0000000000003626 PMC12256081

[6] GAIN Coordinating Center. Global appraisal of individual needs: GAIN-I and GAIN-M90 walkthrough version 5.7. Normal, IL: Chestnut Health Systems; 2016.

[7] Merle JL, Zapata JP, Quieroz A, Zamantakis A, Sanuade O, Mustanski B, Smith JD. Pre-exposure prophylaxis (PrEP) among people who use drugs: a qualitative scoping review of implementation determinants and change methods. Addict Sci Clin Pract. 2024;19(1):46. 38816889 10.1186/s13722-024-00478-2 PMC11138081

[8] Ni Z, Altice FL, Wickersham JA, Copenhaver MM, DiDomizio EE, Nelson LE, Shrestha R. Willingness to initiate pre-exposure prophylaxis (PrEP) and its use among opioid-dependent individuals in drug treatment. Drug Alcohol Depend. 2021;219:108477. 33422864 10.1016/j.drugalcdep.2020.108477 PMC7946167

[9] Woody GE, Bruce D, Korthuis PT, Chhatre S, Poole S, Hillhouse M, Jacobs P, Sorensen J, Saxon AJ, Metzger D, Ling W. HIV risk reduction with buprenorphine–naloxone or methadone: findings from a randomized trial. J Acquir Immune Defic Syndr. 2014;66(3):288–93. 24751432 10.1097/QAI.0000000000000165 PMC4146664

[10] Chan K, Mitchell MM, Casselle E, Bender AA. Facilitators and barriers to PrEP acceptability and initiation among opioid treatment program patients and staff. AIDS Educ Prev. 2024;36(1):60–72. 38349350 10.1521/aeap.2024.36.1.60

[11] Federal Opioid Use. Disorder treatment standards – 42 CFR § 8.12. Available at https://www.law.cornell.edu/cfr/text/42/8.12#:~:text=(i)%20Each%20patient%20admitted%20to,services%20are%20to%20be%20provided. Accessed 24 June 2025.

[12] Jones CM, Byrd DJ, Clarke TJ, Campbell TB, Ohuoha C, McCance-Katz EF. Characteristics and current clinical practices of opioid treatment programs in the united States. Drug Alcohol Depend. 2019;205:107616. 31678836 10.1016/j.drugalcdep.2019.107616

